# An Efficient Hardware Circuit for Spike Sorting Based on Competitive Learning Networks

**DOI:** 10.3390/s17102232

**Published:** 2017-09-28

**Authors:** Huan-Yuan Chen, Chih-Chang Chen, Wen-Jyi Hwang

**Affiliations:** Department of Computer Science and Information Engineering, National Taiwan Normal University, Taipei 116, Taiwan; 80347002s@ntnu.edu.tw (H.-Y.C.); 60447062s@ntnu.edu.tw (C.-C.C.)

**Keywords:** spike sorting, VLSI, competitive learning, brain machine interface

## Abstract

This study aims to present an effective VLSI circuit for multi-channel spike sorting. The circuit supports the spike detection, feature extraction and classification operations. The detection circuit is implemented in accordance with the nonlinear energy operator algorithm. Both the peak detection and area computation operations are adopted for the realization of the hardware architecture for feature extraction. The resulting feature vectors are classified by a circuit for competitive learning (CL) neural networks. The CL circuit supports both online training and classification. In the proposed architecture, all the channels share the same detection, feature extraction, learning and classification circuits for a low area cost hardware implementation. The clock-gating technique is also employed for reducing the power dissipation. To evaluate the performance of the architecture, an application-specific integrated circuit (ASIC) implementation is presented. Experimental results demonstrate that the proposed circuit exhibits the advantages of a low chip area, a low power dissipation and a high classification success rate for spike sorting.

## 1. Introduction

Multi-electrode arrays (MEAs) [1,2] are sensors capable of recording spike data from a large number of neurons of the brain simultaneously. The MEAs have been extensively deployed to facilitate the development of applications such as brain-machine interface (BMI) and/or neuromotor prosthetic devices [3,4] for the rehabilitation of stroke or paralyzed patients. For the MEA-based applications, multi-channel spike sorting [5,6] is usually desired. The spike sorting aims to segregate spikes of individual neurons from the data acquired from each channel. It can be viewed as a clustering process where the spikes belonging to the same neuron are grouped together. The information of the clustering results provided by the spike sorting is essential to the subsequent operations such as neural decoding and control signal generation for prosthetic devices [4].

Data recorded by MEAs may incur a high computational load for spike sorting when real-time operations are necessary. A dedicated hardware circuit offering high computation performance may be beneficial for the acceleration of spike sorting operations. Furthermore, circuits with low area costs and low power density have the additional advantage of bio-implantability for different application needs. Therefore, hardware circuits may be necessary for spike sorting applications where computation speed and bio-implantability are the important concerns.

One hardware approach for the implementation of a spike sorting system is based on the field programmable gate array (FPGA) [7]. A drawback of some FPGA circuits is that they may utilize high area resources and/or dissipate high power. The FPGA systems are then mainly adopted only for offline spike sorting systems [8]. An alternative to the FPGA for hardware implementation is based on the application-specific integrated circuit (ASIC) [9]. The hardware resources and power consumption of some ASIC circuits for spike sorting may be lower than their FPGA counterparts. Therefore, the ASIC implementations may be effective for MEA-based applications in vivo.

A spike sorting system usually provides the operations of spike detection, feature extraction and classification. Many ASIC architectures, however, are not able to offer all these operations. Only the feature extraction and/or spike detection operations are provided in the circuits. Studies in [10,11,12] present ASIC architectures for feature extraction by the principal component analysis (PCA) algorithm and its variants such as the generalized Hebbian algorithm (GHA). Although the PCA-based feature extraction circuits provide feature vectors with high classification success rates, large hardware costs and power consumption may be required for the covariance matrix calculation and eigenvalue decomposition.

To reduce the computational complexities for feature extraction, a number of simple algorithms such as zero crossing [13] and peak detection with area computation (PDAC) [14] are proposed at the expense of a slight degradation in performance as compared with PCA for spike classification. The corresponding PDAC hardware circuits are presented in [14]. Although the architectures have lower area complexities, operations for spike classification are still not included. The incorporation of spike classification may involve online training operations. For the algorithms such K-means [15] and fuzzy C-means (FCM) [16], training in batch mode is necessary. A large memory may be desired to store a training set for the iterative operations of the unsupervised clustering. This may require high area costs for the hardware implementation. Although it is not necessary to store the training set for some incremental clustering algorithms such as online sorting (OSORT) [17], the classification is carried out directly on spike waveforms. Because the algorithm operates without the dimension reduction, the area complexities may still be large [18].

The objective of this paper is to present a novel spike sorting hardware architecture supporting spike detection, feature extraction and classification. The architecture is implemented by ASIC. It is able to achieve accurate classification with low area costs and low power consumption. The spike detection and feature extraction circuits are based on the nonlinear energy operator (NEO) [19] and PDAC algorithms, respectively. The NEO and PDAC have the advantages of simplicity and effectiveness for detection and feature extraction, respectively. To incorporate the hardware classification circuit without incurring a large area overhead, the competitive learning (CL) neural networks [20,21,22] are adopted for spike classification. The CL algorithm is an incremental training algorithm for unsupervised clustering. The CL algorithm and its variants have been found to be effective in a number of clustering applications [22,23,24,25]. It also has been used as a learning vector quantization for supervised learning for spike sorting in [26]. The CL algorithm is well suited for hardware classification operations because it may not be necessary to store training data for the online learning. Furthermore, our experimental results reveal that the classification success rate of the CL may be comparable to that of K-means and FCM for spike sorting.

In the proposed hardware system, the circuits for spike detection, feature extraction and classification can be operated concurrently for different channels. Controllers/buffers are included for the coordination of different units. To accommodate multi-channel spike sorting, dedicated buffers are allocated to each channel for spike detection and feature extraction. Moreover, CL codewords for different channels are stored separately. Nevertheless, computation circuits for detection, feature extraction and classification are shared by all the channels. Furthermore, a clock gating (CG) scheme [27] is adopted so that only the active components acquire the system clock. Both the area costs and power consumption of the proposed circuit can then be minimized. Comparisons with existing hardware architectures confirm that the proposed architecture is an effective alternative for the implementation of implantable multi-channel spike sorting circuit.

The remaining parts of this paper are organized as follows. Section 2 presents the proposed VLSI architecture. The corresponding algorithms are also discussed in detail in this section. The performance evaluation of the proposed circuit is presented in Section 3. Finally, Section 4 includes some concluding remarks.

## 2. The Proposed Circuit

**Figure 1:**
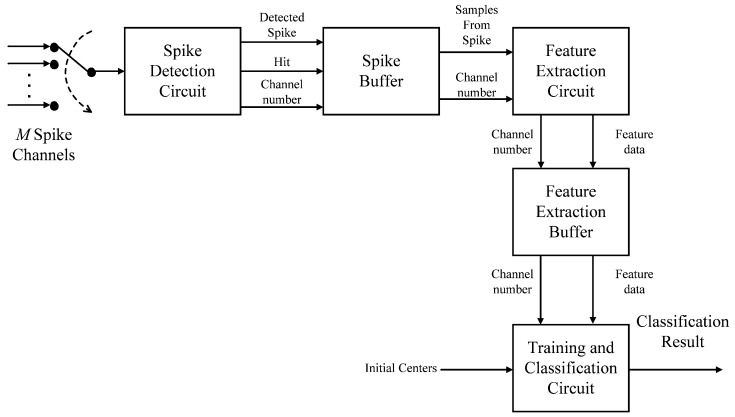
The block diagram of the proposed circuit. It contains five components: spike detection circuit, spike buffer, feature extraction circuit, feature extraction buffer and training and classification circuit.

Figure 1 shows the block diagram of the proposed circuit. The circuit consists of five components: spike detection circuit, spike buffer, feature extraction circuit, feature extraction buffer and training and classification circuit. The spike detection, feature extraction and classification are carried out by the spike detection circuit, the feature extraction circuit and the learning and classification circuit, respectively. A buffer, termed the spike buffer in this work, is adopted for the coordination between the spike detection circuit and feature extraction circuit. Another buffer, termed the feature extraction buffer, is employed for buffering the extracted feature vectors for the subsequent classification and/or online learning operations.

### 2.1. Spike Detection Circuit and Spike Buffer

**Figure 2:**
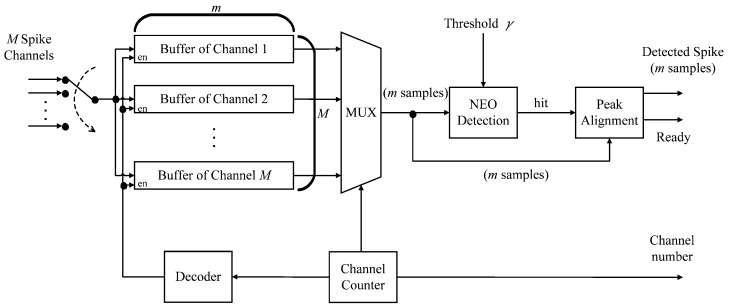
The block diagram of the spike detection circuit. It mainly contains a mixed mode circuit for spike sampling and multiplexing, a channel buffer for storing the spike samples, an NEO detector and a circuit for peak alignment.

The diagram of the spike detection circuit is shown in Figure 2. The circuit supports *M*-channel spike detection by the thresholding operations, where the NEO algorithm is adopted as the preprocessor before thresholding. As shown in Figure 2, the circuit carries out the sampling operations of *M* channels in a round robin fashion. Let $r_{s}$ be the sampling rate for all the channels and $T_{s}=1/r_{s}$ be the sampling period. A mixed mode circuit for the sampling and multiplexing operations is shared by all the channels. It also distributes spike samples one at a time to the channel buffers. During a time interval of $T_{s}$ seconds, the proposed circuit receives *M* samples from the mixed mode circuit. Each is from different channels. Let $r_{c}$ be the clock rate of the circuit and $T_{c}=1/r_{c}$ the clock period. Consequently, *M*, $T_{c}$ and $T_{s}$ are related by:

(1) $$MT_{c}\leq T_{s}.$$

Therefore, the maximum number of channels for a pair of given $T_{c}$ and $T_{s}$, denoted by $M_{\max}$, is given by $M_{\max}=\left\lfloor T_{s}/T_{c}\right\rfloor$.

There are *M* buffers in the circuit. Each channel has its own dedicated buffer. Let *m* be the number of samples of a spike. Each buffer then contains *m* samples. Each of the *M* buffers is a serial-in parallel-out (SIPO) buffer. Among the *M* buffers, only the outputs of one buffer are selected at a time for the NEO detection. The selection is also done in a round robin fashion. This allows all the channels to share the same computation core for spike detection.

Let S(j) be the *j*-th sample of the current channel selected by the multiplexer in the unit for spike detection. The spike detection circuit will issue a “hit” signal when:

(2) $$S^{2}\left(j\right)-S\left(j-1\right)S\left(j+1\right)>\gamma ,$$

for some *j* in the *m* samples of the buffer, where γ>0 is a pre-specified threshold. The selection of γ is based on a scaled version of the standard deviation of training data, as suggested by [28]. Upon receiving the hit signal, the peak alignment circuit will determine the peak of the spike. The *m* samples of that spike after the spike alignment, together with the index of the current channel, are delivered to the spike buffer for subsequent feature extraction operations. For the current channel, let:

(3) $$x\left(n\right)=\left[x_{1},...,x_{m}\right]$$

be the *n*-th spike detected by the spike detection unit. After the detection, the x(n) will then be stored in the spike buffer.

**Figure 3:**
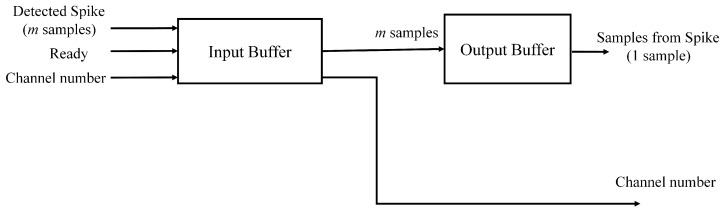
The block diagram of the spike buffer. It consists of the input buffer and output buffer, which are FIFO and parallel-in serial-out (PISO) buffers, respectively. The input buffer stores the detected spikes from the spike detection circuit. The output buffer delivers a detected spike to the feature extraction circuit.

The spike buffer contains two stages, as shown in Figure 3. The buffer at the first stage (termed the input buffer in Figure 3) is an *m*-sample input, *m*-sample output first-in-first-out (FIFO) buffer. The buffer is able to store up to *M* spikes from different channels. The input buffer is triggered by the “ready” signal issued from the spike alignment circuit. After the triggering, the input buffer stores the *m* samples presented in the input port, together with the corresponding channel index. The buffer at the second stage is a parallel-in serial-out (PISO) buffer. It is termed the output buffer in Figure 3. The goal of the output buffer is to fetch the spikes stored in the input buffer, together with the corresponding channel indices, and then deliver them to the feature extraction unit. The output buffer is able to read a spike *m* samples at a time from the input buffer. Because the feature extraction unit supports only serial transmission, the output buffer sends the spike to the feature extraction unit on a sample-by-sample basis.

### 2.2. Feature Extraction Unit and Feature Extraction Buffer

The feature extraction unit is shared by all the *M* channels. The unit computes the feature vectors of spikes one at a time. In the proposed architecture, the PDAC algorithm in [14] is adopted for the feature extraction. Given a spike x(n) in Equation (3) provided by the spike buffer, the feature extraction unit first computes $a_{1}\left(n\right)$ and $a_{2}\left(n\right)$, defined as:

(4) $$\begin{array}{ccc} a_{1}\left(n\right) & = & \sum_{i=1}^{i_{\min}}\left(x_{i}-x_{i_{\min}}\right), \end{array}$$

(5) $$\begin{array}{ccc} a_{2}\left(n\right) & = & \sum_{i=i_{\min}+1}^{m}\left(x_{i}-x_{i_{\min}}\right), \end{array}$$

where:

(6) $$i_{\min}=\underset{1\leq i\leq m}{\mathrm{argmin}}x_{i}.$$

**Figure 4:**
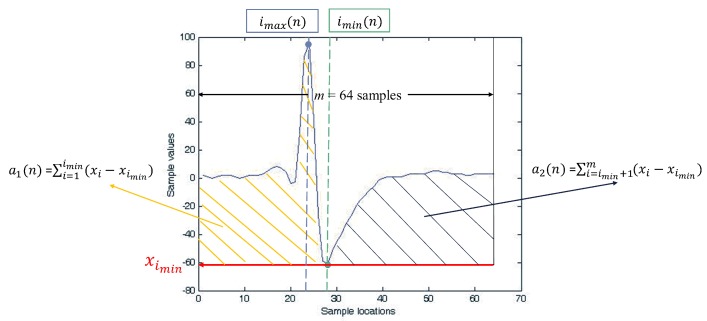
An example of the feature extraction computation of the PDAC algorithm. The $a_{1}\left(n\right)$ and $a_{2}\left(n\right)$ are the areas of the shaded regions shown in the figure. After $a_{1}\left(n\right)$ and $a_{2}\left(n\right)$ are obtained, the features $f_{1}\left(n\right)$ and $f_{2}\left(n\right)$ are obtained by Equation (8).

Therefore, $a_{1}\left(n\right)$ and $a_{2}\left(n\right)$ are two non-overlapping areas of x above $x_{i_{\min}}$, separated by $i_{\min}$. An example of $a_{1}\left(n\right)$ and $a_{2}\left(n\right)$ for a spike x is shown in Figure 4. In the PDAC algorithm, the feature vector associated with x(n), denoted by X(n), is computed by:

(7) $$X\left(n\right)=[f_{1}\left(n\right),f_{2}\left(n\right)],$$

where:

(8) $$f_{i}\left(n\right)=a_{i}\left(n\right)/\left(i_{\min}-i_{\max}\right),\;i=1,2,$$

and:

(9) $$i_{\max}=\underset{1\leq i\leq m}{\mathrm{argmax}}x_{i}.$$

**Figure 5:**
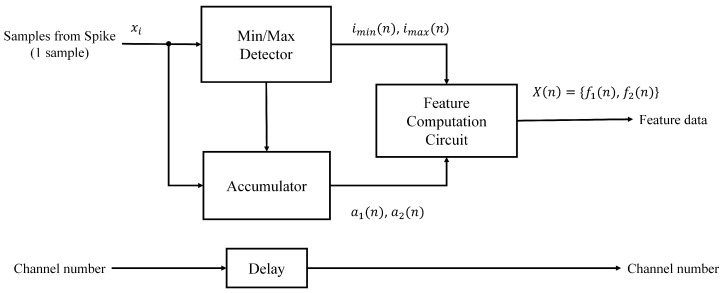
The block diagram of feature extraction circuit. It contains the min/max detector, accumulator and feature computation circuit. Both the min/max detector and accumulator are operated concurrently to expedite the computation.

While providing feature vectors with high classification accuracy [14], the PDAC algorithm is computationally efficient. Figure 5 shows the architecture of the feature extraction unit based on PDAC. It contains three modules: an accumulator, a min/max detector and a feature computation circuit. The goal of the accumulator is to compute $a_{1}$ and $a_{2}$ in Equations (4) and (5). The min/max detector is used to find $i_{\min}$ and $i_{\max}$. Based on $a_{i},i=1,2,$$i_{\min}$ and $i_{\max}$, the feature computation circuit then produces $f_{i},i=1,2,$ by Equation (8).

Both the accumulator and min/max detector are operated one sample at a time. Although the computation of $a_{i},i=1,2,$ is dependent on $i_{\min}$, a remarkable fact of the PDAC algorithm is that the accumulator and min/max detector can still be operated concurrently [14]. Furthermore, in the PDAC algorithm, no pre-processing or training processes are required. By contrast, they are necessary for PCA and its variant GHA. All these advantages are beneficial for attaining the fast computation time and low area costs.

After the feature extraction operations are completed, the resulting feature vector X(n) and its associated channel index are stored in the feature extraction buffer for subsequent training and/or classification operations. The feature extraction buffer is an FIFO buffer containing a fixed number of stages. Its output port is connected to the training and classification circuit.

### 2.3. Training and Classification Circuit

**Figure 6:**
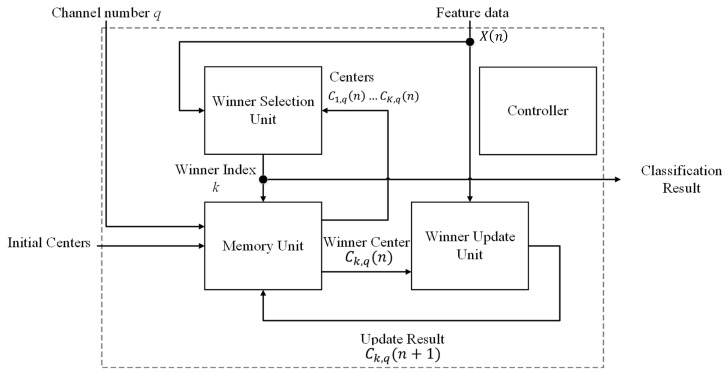
The block diagram of the training and classification circuit. In addition to the controller, there are three modules in the circuit: winner selection unit, memory unit and winner update unit. All the modules are used for the CL online training. Only the winner selection unit and memory unit are used for the spike classification.

The architecture of the training and classification unit is shown in Figure 6. It contains the winner selection unit, the memory unit, the winner update unit and the controller. The winner selection unit, memory unit and winner update unit are all involved in the CL-based online-training for the spike clustering. After the CL training process is completed, only the winner selection unit and memory unit are used for the spike classification. The controller is used for the coordination of these units. In the proposed architecture, all the *M* channels share the same winner selection unit and winner update unit for CL training and classification. Furthermore, each channel has its own entries in the memory unit for storing the CL training results of that channel. In this way, the area costs can be effectively reduced.

Because the training process is based on the CL algorithm, before discussing the operations of the circuit, we first present the CL algorithm. Let *K* be the number of clusters for the spike clustering. Let $C_{p,q}$ be the center of the *p*-th cluster,p=1,...,K, for the spikes from the *q*-th channel, q=1,...,M. The CL algorithm operates in an incremental manner for the training operations for each channel. The centers associated with each cluster of a channel may be updated during the online training process when a spike from that channel is detected, and its feature vector is extracted.

Suppose x(n) is the *n*-th spike detected from the channel *q* by the spike detection circuit and X(n) is its feature vector computed by the feature extraction unit. In the CL algorithm, X(n) is used for the updating of cluster centers. Let $C_{p,q}\left(n\right)$ and $C_{p,q}\left(n+1\right)$ be the center of the *p*-th cluster before and after the updating by X(n), where $C_{p,q}\left(0\right)$ is the initial cluster center. They can be obtained by random selection from the training set. Moreover, let $C_{k,q}\left(n\right)$ be the cluster center closest to X(n) among all the centers $C_{p,q}\left(n\right),p=1,...,K$. That is, the index *k* of $C_{k,q}\left(n\right)$ should satisfy:

(10) $$k=\underset{1\leq p\leq K}{\mathrm{argmin}}d\left(C_{p,q}\left(n\right),X\left(n\right)\right),$$

where $d(C_{p,q}\left(n\right),X\left(n\right))$ is the squared distance between $C_{p,q}\left(n\right)$ and X(n). We call $C_{k,q}\left(n\right)$ the winner of the competition. After the winner is identified, the winner-take-all updating scheme is then carried out. In the scheme, the $C_{p,q}\left(n\right)$ and $C_{p,q}\left(n+1\right)$ are related by:

(11) $$\begin{array}{ccc} C_{k,q}\left(n+1\right) & = & C_{k,q}\left(n\right)+\eta \left(X\left(n\right)-C_{k,q}\left(n\right)\right), \end{array}$$

(12) $$\begin{array}{ccc} C_{p,q}\left(n+1\right) & = & C_{p,q}\left(n\right),\;\mathrm{when}\;p\neq k, \end{array}$$

where η>0 is the learning rate. Algorithm 1 shows the CL algorithm for the on-line training of spikes from channel *q*. It is clear from Algorithm 1 that an advantage of the CL algorithm is its simplicity. It is not necessary to store the training vectors. They can be obtained online and then immediately used for the incremental updating of cluster centers. We can view the cluster centers after updating as the current training results of the CL algorithm.

**Algorithm 1** CL algorithm for spike clustering of channel *q* by *N* training spikes
**Require:** Learning rate η.**Require:** Number of training spikes *N*.**Require:** Initial centers $C_{p,q}\left(0\right),p=1,...,K.$1:**for** n=0 to N-1 **do**2: Obtain X(n) from the feature extraction buffer.3: Compute *k* from X(n) and $C_{p,q}\left(n\right),p=1,...,K,$ by (10).4: Update $C_{p,q}\left(n+1\right),p=1,...,K,$ by (11) and (12).5:**end for**


The major advantage of the CL algorithm is the low computational complexity. When the number of classes *K* is known and the dimension of the feature vector is two, the computational complexity of CL and K-means during the training phase is O(2KT) and O(2KTt), respectively, where *T* is the number of training vectors and *t* the number of iterations for K-means. The K-means algorithm may have higher computational complexity because it operates in an iterative fashion. For the FCM algorithm, the computational complexity would become $O(2K^{2}Tt)$ because of the requirement for the computation of membership functions. The computational complexity of OSORT is O(mKT). Because the number of samples of a spike *m* is usually larger than two, the OSORT also has higher computational complexity than that of CL.

**Figure 7:**
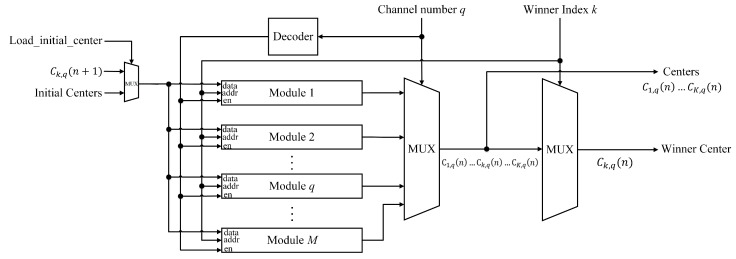
The block diagram of the memory unit. There are *M* modules in the circuit, where the module q,q=1,...,M, is dedicated to channel *q* for CL training and classification. Each module contains *K* centers associated with its dedicated channel.

In the proposed CL circuit, the cluster centers for each channel are stored in the memory unit of the training and classification circuit shown in Figure 7. The circuit contains *M* modules, where each module is dedicated to a different channel. The module *q* stores the cluster centers associated with channel *q*. As shown in Figure 7, the circuit is able to provide *K* centers $C_{p,q}\left(n\right),p=1,...,K,$ of a channel specified by the channel number *q* in parallel. All the *K* centers can be used for the winner competition process for CL learning or the classification process after learning. During the learning phase, the circuit is also able to provide the winner $C_{k,q}\left(n\right)$ after the competition process is completed and the winner index *k* is available. Furthermore, after the winner is identified and the corresponding center is updated, the circuit is able to store the updated center $C_{k,q}\left(n+1\right)$ to the corresponding module specified by the channel number *q*.

**Figure 8:**
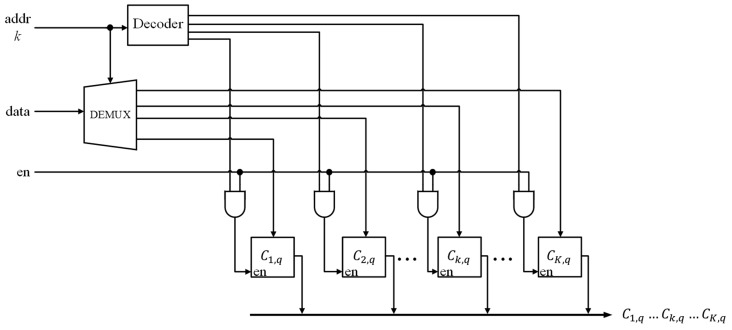
The block diagram of each module of the memory unit. It consists of *K* cells, where each cell stores a center associated with its dedicated channel.

Figure 8 shows the architecture of each module of the memory unit. It can be observed from the figure that there are *K* cells in each module. The content of the *K* cells can be fetched concurrently for winner competition and classification. Nevertheless, because of the winner-take-all updating scheme, only one of the cells will be updated, which is specified by the winner index *k*.

**Figure 9:**
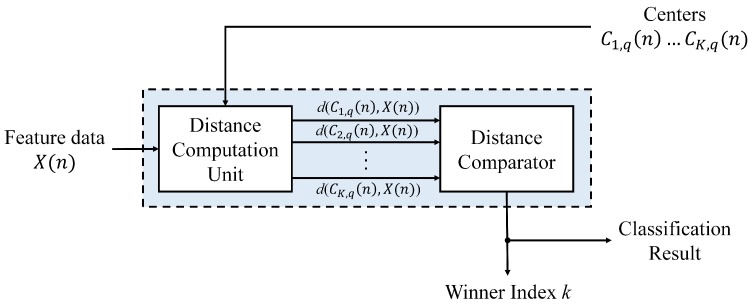
The block diagram of the winner selection unit. There are two modules in the circuit: distance computation unit and distance comparator. The circuit fetches *K* centers associated with a channel *q* and produces the classification results and winner index.

Based on the cluster centers stored in the memory unit, the winner selection unit in the proposed training and classification circuit can be employed for winner competition or classification. When the proposed circuit operates in CL training mode, the output of the circuit indicates the index of the winner. Otherwise, it reveals the class to which the detected spike belongs. Figure 9 shows the architecture of the winner selection unit. It contains two components: the distance computation unit and the comparator. The distance computation unit computes the squared distance $d(C_{p,q}\left(n\right),X\left(n\right))$ in parallel for all p,p=1,...,K. The unit contains adders and multipliers for the distance calculation. The computation results are then delivered to the comparator for finding *k* satisfying Equation (10).

**Figure 10:**
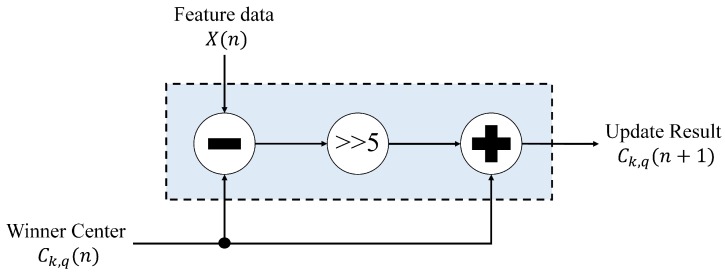
The architecture of winner update unit. The value of learning rate is $\eta =2^{-5}$ in the circuit.

After winner index *k* is identified by the winner selection unit, the winner updated unit is activated to compute $C_{k,q}\left(n+1\right)$ from $C_{k,q}\left(n\right)$ by Equation (11), where $C_{k,q}\left(n\right)$ is provided by the memory unit. The architecture of the winner update unit is revealed in Figure 10, which contains only adders and a shift operator. From Equation (11), it follows that multiplication may be desired to take the learning rate η into account for the CL training. Nevertheless, by restricting the learning rate to be a power of two, the multiplication can be reduced to a shift operation. This is beneficial for the reduction of the area costs. After the computation is completed, the updated center, $C_{k,q}\left(n+1\right)$, is then delivered back to the memory unit. This completes the training process for the feature vector X(n).

The goal of the controller of the training and classification circuit is to coordinate different components of the circuit depending on its operation mode. When the circuit is in the classification mode, only the memory unit and winner selection unit are activated by the controller. In this mode, the memory unit first provides centers $C_{p,q}\left(n\right),p=1,...,K,$ to the winner selection unit. According to the centers and the input feature vector X(n), the winner selection unit produces the class index *k*. When the circuit is in the CL training mode, there are two phases. The first phase is identical to the classification mode, where the output *k* is regarded as the winner index. In the second phase, the controller activates the memory unit and winner update unit. After the completion of this phase, the updated center $C_{k,q}\left(n+1\right)$ is obtained. This completes the training for one iteration. The controller may continue the CL training until a sufficient number of iterations has been carried out for each channel.

An advantage of the proposed training and classification circuit is that it provides a high degree of flexibility for the online training. Because it is based on CL, the training process is inherently incremental. It is not necessary to store training data before training operations. The spikes recorded by MEAs can be directly used as the training data, and there is no need to retain them after they have been used by the circuit. Because no dedicated memory circuits for storing the training sets are required, there will be no constraints on the size of training sets. When high classification accuracy is desired, larger training sets may be necessary at the expense of a longer training time for collecting training data from MEAs. Alternatively, smaller training sets can be adopted for a shorter training time.

The flexibility of the CL algorithm also allows the number of clusters *K* to be determined adaptively. Although *K* can be pre-specified before the training, an OSORT-like technique can be adopted to compute *K* adaptively during the training process. Starting from K=2, the algorithm compares the distance between the current training vector and its closest center. If the distance is above a threshold, a new cluster may be created to accommodate the current training vector. In this case, the controller in the architecture shown in Figure 6 is responsible for the determination of *K*.

Finally, due to the flexibility, it is possible to extend the proposed circuit to the spike trains acquired by tetrodes. In this case, the circuit contains four channels for each tetrode. Each channel has dedicated spike detection and feature extraction circuits. Because the spikes from the four channels can be jointly trained and classified [29], they can share the same CL training and classification circuit. The area costs in this case therefore may be lower than those of its MEA counterpart.

## 3. Experimental Results

We evaluate the performance of the proposed circuit in this section. Because the area of the circuit is dependent on the numbers of hardware arithmetic operators and resisters, they are considered as the area complexities of the circuit. The area complexities are presented as the big O function, describing the asymptotic behavior of the complexities. The training and classification circuit is the most important part of the circuit. Therefore, the area complexities of the training and classification circuit are first considered, as shown in Table 1. We can observe from Table 1 that all types of area costs grow with the number of classes *K*. Only the number of registers increases with the number of channels *M*. The area complexities for arithmetic operations are not dependent on *M* because all the channels share the same computation core for CL training and classification.

In addition to the training and classification circuit, the area complexities of the other circuits are also independent of *M*. Table 2 reveals the area complexities of all the circuits in the proposed architecture. As shown in the figure, the number of hardware arithmetic operators is dependent on the number of classes *K* and is independent of the number of channels *M* for all the circuits. As *M* increases, only the number of registers grows in these circuits.

The actual area of the proposed circuit is also considered. We carry out the ASIC implementation of the circuit for the measurement of the area. The Taiwan Semiconductor Manufacturing Company (TSMC) 90-nm technology is adopted for the implementation. The Synopsys Design Compiler is used as the platform for gate level design. The dimension of spikes is set to m=64. The impact of the number of classes *K* and the number of channels *M* on the area (μm${}^{2}$) is revealed in Table 3. We can see from the table that the area increases with *K* and *M*. These facts are consistent with those revealed in Table 2.

To further study the impact of the number of channels *M* on the area costs, the normalized area per channel is considered. We define the normalized area of a circuit as the total area of the circuit divided by the number of channels *M*. The normalized area can be viewed as the average area cost per channel. Table 4 shows the corresponding results. From Table 4, it can be concluded that the normalized area decreases as *M* increases. This is because spike sorting for different channels in the proposed circuit shares the same computation cores. Because of the hardware resource sharing implementation, better efficiency in area costs for a larger *M* is observed.

Although the area of some components of the proposed circuit are dependent on the number of channels *M*, their dependency may vary. Table 5 shows the area of each component of the proposed circuit for different numbers of channels *M*. The number of classes *K* is three. It can be observed from the table that the spike detection circuit and spike buffer have high growth rates in area for the number of channels *M*. In fact, when *M* increases from 2–64, the percentage of the area of the spike detection circuit out of total area of the proposed circuit grows from 25–45%. Similarly, when M=64, the spike buffer consumes 43% of the total area of the proposed circuit. On the contrary, the training and classification circuit only consumes 11% for M=64.

Both the spike detection circuit and spike buffer have high areas when the number of channels is large because the circuits need to store sampled data and detected spikes for the subsequent operations. The length of the sampled data for each channel in the spike detection circuit is m=64. Furthermore, the length of detected spikes is also m=64 in the spike buffer. Therefore, the increment in area would be large. On the contrary, in the training and classification circuit, it is only necessary to store K=3 centers for each channel. The increment in area by increasing *M* in the training and classification circuit may then be smaller than that in the spike detection circuit and spike buffer. The area of the feature the extraction circuit is independent of channel number *M*. The percentage of the area of the feature extraction circuit out of the total area therefore decreases with *M*. In particular, when M=64, the feature extraction circuit consumes only 1% of the total area, as shown in Table 5.

Another important performance measurement of a circuit is the power consumption. Table 6 reveals the normalized power consumption of the proposed circuit for various numbers of channels *M*, where the normalized power (mW per channel) is defined as the division of the total power (mW) of the circuit by *M*. In the experiment, the clock rate $r_{c}$ is 1 MHz. Synopsis Prime Time is employed as the CAD tool for the power consumption measurement. Note from Table 4 that the normalized area decreases for larger numbers of channels *M*. Therefore, it can be observed from Table 6 that the proposed circuit has smaller normalized power consumption for larger *M*. Moreover, from Table 6, we see that the power consumption can be further reduce by the employment of clock gating (CG). This is because the dynamic power consumption of inactive components of the circuit can be reduced by not supplying the clock signal to the components. The reduction in power (as a percentage) due to the employment of CG is also included in Table 6. When M=64, we see that a 41% reduction in normalized power consumption can be achieved for the proposed circuit operating with CG. In addition to area and power consumption, the power density may also be an important concern. Studies in [30] recommend that the power density should be below 80 mW/cm${}^{2}$ to avoid potential brain damage. In the proposed circuit with CG, the power density is 68.35 mW/cm${}^{2}$ for M=64 and K=3. The circuit may then be effective as an implantable device.

After evaluating the area and power consumption, we next compare the proposed circuit with other ASIC architectures for spike sorting in Table 7. In the proposed implementation, only the case that the number of channels M=64 and the number of classes K=3 is considered. A direct comparison of these ASIC circuits may be difficult because they may be the hardware implementation of different spike sorting algorithms. Moreover, they may be based on different technologies and operating clock rates. However, it can be seen from Table 7 that some existing implementations [11,12,14] do not support learning or classification functions. Furthermore, as compared with implementations offering online classification, the proposed architecture has superior area-power performance. In particular, the normalized area of the proposed architecture is lower than those of [10,18]. This is because the PCA algorithm used by [10] may impose high area costs for constructing the covariance matrix. Furthermore, the OSORT classification technique adopted by [18] carries out the classification directly on waveforms without dimension reduction. A large memory may be required for storing the waveforms, resulting in large area and/or power consumption.

**Figure 11:**
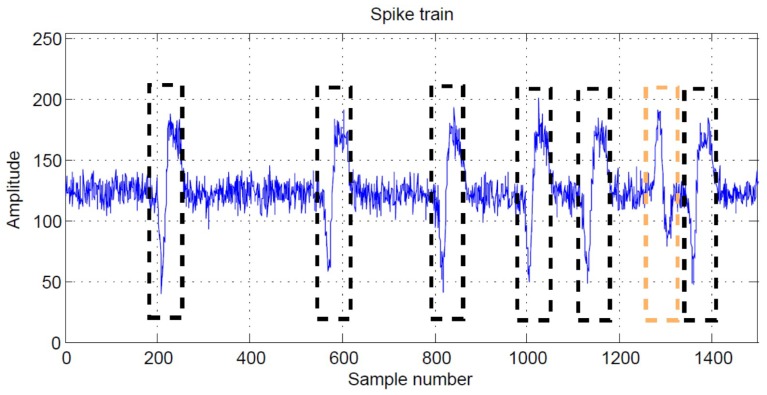
A segment of the waveform of a spike train produced by the simulator in [31]. There are two classes of spikes (i.e., K=2). Spikes belonging to the first class and the second class are marked by black rectangles and orange rectangles, respectively.

Although the proposed architecture has low area costs, it is effective for spike classification. The comparisons of the classification success rate (CSR) of the CL algorithm, K-means algorithm [15], FCM algorithm [16] and OSORT algorithm [17] for spike sorting are shown in Table 8. The feature vectors for the clustering are produced by the PCA [10], GHA [12] and PDAC [14] algorithms for the CL algorithm, K-means algorithm and CL algorithm. We define the CSR as the division of the number of spikes receiving correct classification by the total number of spikes. The spike trains for the experiments in Table 8 are obtained from the database provided in [28] and the simulator developed in [31], respectively. For the spike trains acquired from the database in [28], the number of classes is K=3. They are labeled by the file names: C_Easy1_noise01, C_Easy2_noise01 and C_Difficult2_noise005, respectively. For the spike trains obtained by the simulator in [31], they are specified in terms of the SNR levels: 1 dB, 4 dB, 6 dB and 8 dB, respectively. The number of classes is K=2. The ground truth about the spiking activity for all the spike trains can be accessed for CSR assessment. An example of a spike train with two classes produced by the simulator in [31] with SNR = 8 dB is shown in Figure 11.

Based on the feature vectors produced from the same feature extraction algorithm, it can be observed from Table 8 that the CL algorithm has comparable CSR to that of the K-means and FCM algorithms. For example, for the waveforms from “C_Easy2_noise01” in the database in [28], we can see from Table 8 that the CSRs of the CL, K-means and FCM are 96.65%, 96.70% and 96.68% for the feature vectors produced by the PCA algorithm, respectively. Furthermore, for the spike trains with SNR = 1 dB shown in Table 8, the CSRs of the CL, K-means and FCM are 95.71%, 95.92% and 96.32% for the feature vectors produced by the PDAC algorithm, respectively. For the same sources, the CL, K-means and FCM also attain CSRs of 99.70%, 99.79% and 99.73% for the feature vectors produced by PCA, respectively.

**Figure 12:**
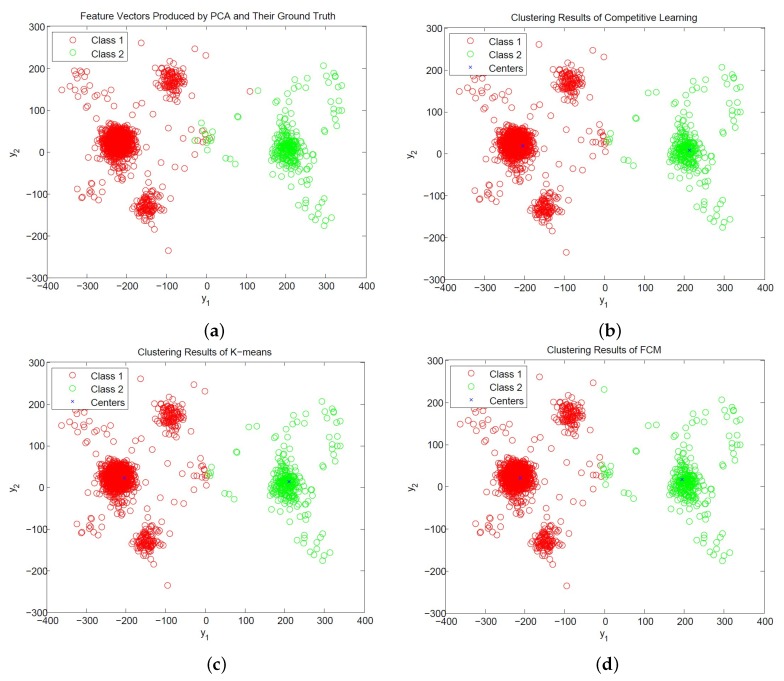
The distribution of PCA feature vectors of spikes and the results of CL, K-means and FCM clustering for SNR = 1 dB. (**a**) Ground truth of neuron spikes; (**b**) clustering results produced by CL; (**c**) clustering results produced by K-means; (**d**) clustering results produced by FCM.

**Figure 13:**
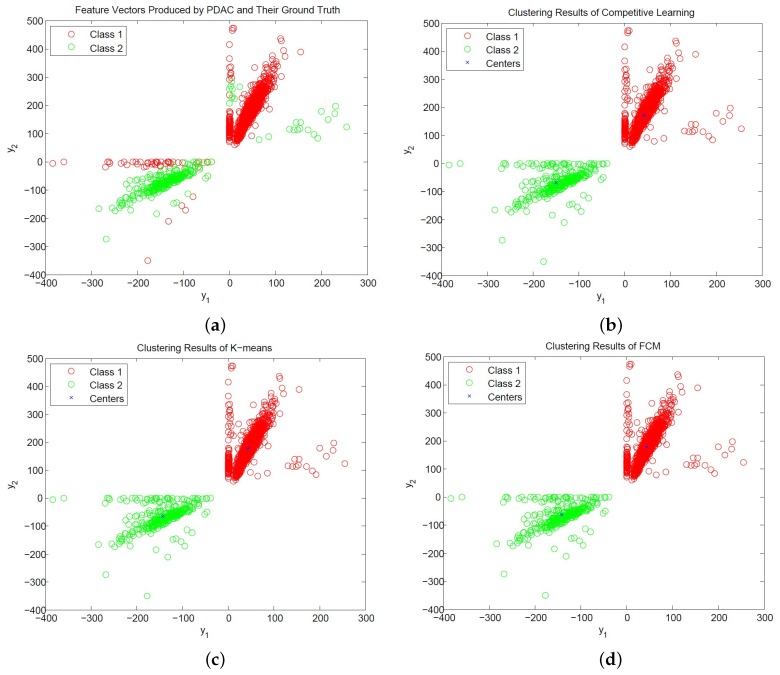
The distribution of PDAC feature vectors of spikes and the results of CL, K-means and FCM clustering for SNR = 1 dB. (**a**) Ground truth of neuron spikes; (**b**) clustering results produced by CL; (**c**) clustering results produced by K-means; (**d**) clustering results produced by FCM.

Figure 12 and Figure 13 show the distribution of PCA and PDAC feature vectors of the spike trains form [31] with SNR = 1 dB and the classification results of the CL, K-means and FCM algorithms. We can observe from the figures that all the algorithms produce similar centers for classification for PCA and PDAC feature vectors. Therefore, they have similar CSR values. Because the CL algorithm is based on incremental training operations, its hardware implementation may impose less resource consumption than its batch training counterparts such as K-means and FCM. Therefore, the CL algorithm may be an effective alternative for the hardware implementation of spike classification.

From Table 8, we can also observe that the employment of PDAC and CL (i.e., PDAC + CL) has comparable CSR to other combinations of feature extraction and classification techniques. In particular, for the spike trains with SNR = 1 dB shown in Table 8, the CSRs of PDAC + CL, PCA + FCM and OSORT are 95.71%, 99.73% and 99.37%, respectively. The combination of PDAC and CL produces only a small degradation in CSR as compared with its counterpart with PCA + FCM and OSORT.

**Figure 14:**
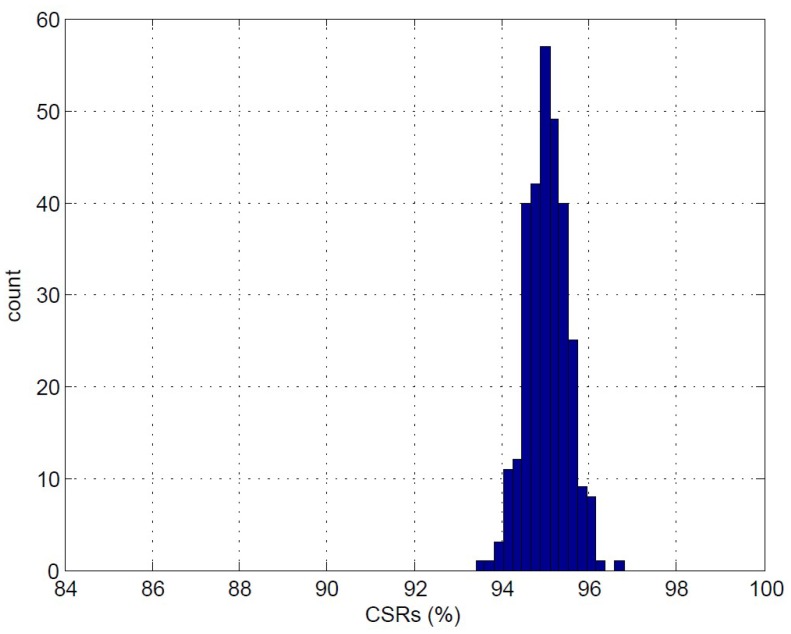
The distribution of the CSRs of 300 independent clustering operations for K=2 based on the feature vectors produced by the PDAC algorithm. The training vectors are produced by the simulator [31] with SNR = 1 dB.

The performance of the CL may be dependent on the selection of the initial centers. Therefore, it would be beneficial to investigate the robustness of the CL algorithm to the initial centers for the spike sorting. Figure 14 reveals the distribution of CSRs of 300 independent CL clustering operations for K=2 based on the feature vectors produced by the PDAC algorithm. The training vectors are produced by the simulator [31] with SNR = 1 dB. Each clustering operation is based on initial centers randomly selected from the same training set. It can be observed from Figure 14 that all the CL clustering operations result in similar CSRs even with different initial centers. In fact, the CSR values are concentrated in a small interval ranging from 94.8–97.0%. The robustness of the CL to the selection of initial centers is beneficial because the random selection of initial centers from the MEA-recorded spikes may be sufficient for the training. All these results reveal the effectiveness of the proposed architecture.

## 4. Conclusions

We have implemented the proposed architecture for spike sorting by ASIC with 90-nm technology. The architecture supports spike detection, feature extraction and classification, which are based on the NEO, PDAC and CL algorithms, respectively. The algorithms have the advantages of effectiveness and simplicity for the hardware implementation. When the number of channels is 64, the normalized area of the proposed architecture is 0.0266 mm${}^{2}$/ch, which is lower than that of the other implementations considered in this paper supporting spike classification. The proposed circuit has a normalized power dissipation of 18.18 μW/channel when the clock rate is 1 MHz. The CL algorithm also has CSR values comparable to those of the K-means and FCM algorithms. Consequently, the proposed circuit exhibits the advantages of low hardware resource and power consumption and high classification accuracy for the implementation of implantable multi-channel spike sorting systems.

## Figures and Tables

**Table 1:** The area complexities of the training and classification circuit.

	Comparators	Adders/Subtractors	Multipliers/Dividers	Registers
Memory Unit	0	0	0	O(MK)
Winner Selection Unit	O(K)	O(K)	O(K)	O(K)
Winner Update Unit	0	O(1)	0	O(1)
Total	O(K)	O(K)	O(K)	O(MK)

**Table 2:** The area complexities of the proposed architecture.

	Comparators	Adders/Subtractors	Multipliers/Dividers	Registers
Spike Detection Circuit	O(1)	O(1)	O(1)	O(Mm)
Spike Buffer	0	0	0	O(Mm)
Feature Extraction Circuit	O(1)	O(1)	O(1)	O(1)
Training and Classification Circuit	O(K)	O(K)	O(K)	O(MK)
Total	O(K)	O(K)	O(K)	O(MK+Mm)

**Table 3:** The area (μm${}^{2}$) of the proposed circuit for different numbers of channels *M* and classes *K*.

Number of Classes *K*	Number of Channels *M*					
	2	4	8	16	32	64
2	82,667	133,402	233,766	433,482	834,448	1,644,525
3	93,355	145,429	248,966	455,372	869,329	1,705,028
4	103,675	157,539	264,414	477,274	903,921	1,766,590

**Table 4:** The normalized area per channel (μm${}^{2}$/channel) of the proposed circuit for different numbers of channels *M* and classes *K*.

Number of Classes *K*	Number of Channels *M*					
	2	4	8	16	32	64
2	41,333	33,350	29,220	27,092	26,076	25,695
3	46,677	36,357	31,120	28,460	27,166	26,641
4	51,837	39,384	33,051	29,829	28,247	27,602

**Table 5:** The area (μm${}^{2}$) of each component of the proposed circuit for different numbers of channels *M*.

Number of	Number of	Spike Detection	Spike	Feature Extraction	Training and	Total
Channels *M*	Classes *K*	Circuit	Buffer	Circuit	Classification Circuit		
2	3	23,149 (25%)	23,458 (25%)	14,524 (16%)	32,224 (34%)	93,355
4	3	47,498 (33%)	46,611 (32%)	14,524 (10%)	36,796 (25%)	145,429
8	3	95,359 (38%)	92,674 (37%)	14,524 (6%)	46,409 (19%)	248,966
16	3	189,931 (42%)	185,079 (41%)	14,524 (3%)	65,838 (14%)	455,372
32	3	380,117 (44%)	370,015(42%)	14,524 (2%)	104,673 (12%)	869,329
64	3	759,136 (45%)	749,894 (43%)	14,524 (1%)	181,474 (11%)	1,705,028

**Table 6:** The normalized power consumption per channel (μW/channel) of the proposed circuit for various numbers of channels *M*.

Number of	Number of	Clock Rate	Normalized Power		Power
Channels *M*	Classes *K*	$r_{c}$	Without CG	With CG	Reduction
2	3	1 MHz	59.85	45.64	24%
4	3	1 MHz	44.70	31.27	30%
8	3	1 MHz	37.56	24.42	35%
16	3	1 MHz	33.66	20.82	38%
32	3	1 MHz	31.65	18.91	40%
64	3	1 MHz	30.70	18.18	41%

**Table 7:** Comparisons of various ASIC implementations for spike sorting.

	Number of	Power	Area	Technology	Spike	Feature	Classification
	Channels	(μW/ch.)	(mm${}^{2}$/ch.)		Detection	Extraction		
[10]	16	256.875	1.770	350 nm	NEO	PCA	Table look-up
[11]	16	8.59	0.268	130 nm	No	SPIRIT	No
[12]	64	85.82	0.0805	90 nm	NEO	GHA	No
[14]	64	20.53	0.0211	90 nm	NEO	PDAC	No
[18]	1	14.60	0.077	45 nm	Thresholding	No	OSORT
Proposed	64	18.18	0.0266	90 nm	NEO	PDAC	CL

**Table 8:** The classification success rates (CSRs) (%) of various feature extraction and classification algorithms for spike sorting. The spike trains are obtained from the database in [28] or by the simulator in [31]. For the spike trains acquired from the database in [28], they are labeled by the file names: C_Easy1_noise01, C_Easy2_noise01 and C_Difficult2_noise005, respectively. For the spike trains obtained by the simulator in [31], they are specified in terms of the SNR levels: 1 dB, 4 dB, 6 dB and 8 dB, respectively.

Algorithms		Database in [28]			Simulator in [31]			
		C_Easy1	C_Easy2	C_Difficult2	SNR			
		_noise01	_noise01	_noise05	1 dB	4 dB	6 dB	8 dB
	CL	99.32	96.65	98.60	99.70	99.76	99.82	99.82
PCA	K-means	99.32	96.70	98.57	99.79	99.80	99.76	99.76
	FCM	99.32	96.68	98.57	99.73	99.75	99.82	99.88
	CL	99.32	94.12	82.02	99.82	99.83	99.86	99.74
GHA	K-means	99.32	94.26	81.51	99.76	99.82	99.70	99.76
	FCM	99.32	94.35	81.78	99.80	99.82	99.82	99.81
	CL	93.38	90.57	83.06	95.71	96.56	96.75	96.67
PDAC	K-means	93.38	90.57	82.85	95.92	95.77	96.73	96.65
	FCM	93.36	90.43	83.03	96.32	96.45	96.71	96.73
OSORT		99.32	98.01	98.72	99.37	99.06	99.58	99.68

## Abbreviations

The following abbreviations are used in this manuscript:

ASIC	Application-specific integrated circuit
BMI	Brain machine interface
CG	Clock gating
CL	Competitive learning
FCM	Fuzzy C-means
FIFO	First-in-first-out
FPGA	Field programmable gate array
GHA	Generalized Hebbian algorithm
MEA	Multi-electrode array
NEO	Nonlinear energy operator
OSORT	Online sorting
PCA	Principal component analysis
PDAC	Peak detection with area computation
SPIRIT	Streaming pattern discovery in multiple time-series
TSMC	Taiwan Semiconductor Manufacturing Company
